# ST analysis of the fetal electrocardiogram – Comments on recent experimental data

**DOI:** 10.1371/journal.pone.0221210

**Published:** 2019-08-22

**Authors:** Ingemar Kjellmer, Kaj Lindecrantz, Karl G. Rosén

**Affiliations:** 1 Department of Pediatrics, Institute of Clinical Sciences, Sahlgren´s Academy, University of Gothenburg, Gothenburg, Sweden; 2 School of Technology and Health, Royal Institute of Technology, Stockholm and Department of Clinical Science, Intervention and Technology, Karolinska Institutet, Stockholm, Sweden; 3 Department of Physiology, Sahlgren´s Academy, University of Gothenburg, Gothenburg, Sweden; University of Washington, UNITED STATES

## Abstract

In their paper, Andriessen at al present a validation of fetal ECG analysis and the clinical STAN device in midgestation fetal lambs exposed to 25 minutes of umbilical cord occlusion. The study presents results that contrast remarkably from previously published experimental data which raises a number of questions and comments.

The most striking finding of Andriessen et al is the recording of an extremely high number of alarms from the STAN equipment during control conditions when no alarms at all are expected. These patterns have never been seen, neither in the clinical situation nor in our own fetal sheep studies. The reason for this becomes apparent when their way of recording the FECG is scrutinized. In their assessment of STAN, Andriessen at al use an assumed negative aVF lead with the assumption that it will reflect the FECG in the same way as the unipolar scalp lead used clinically. The signal used for disqualification of STAN is itself not qualified to properly represent the fetal scalp lead signal that STAN is designed for. To question a proven technology is fully accepted but those attempting would be asked to argue along fully validated data and related analysis including questioning of their own data.

## Introduction

Our ability to make an accurate assessment of fetal well-being during labour is a great challenge. Animal and human studies have shown that fetal hypoxemia during labour can alter the shape of the fetal electrocardiogram (ECG) waveform, notably elevation of the T-wave and alterations of the ST segment. A medical device (STAN^®^, Neoventa Medical, Moelndal, Sweden) has been developed to monitor the fetal ECG during labour as an adjunct to continuous electronic fetal heart rate monitoring (CTG+ST analysis; STAN).

In their paper, Andriessen at al present a validation of fetal ECG analysis and the clinical STAN device in midgestation fetal lambs exposed to 25 minutes of umbilical cord occlusion [1]. The study presents results that contrast remarkably from previously published experimental data which raises a number of questions and comments.

The most striking finding of Andriessen et al is the recording of an extremely high number of alarms from the STAN equipment during control conditions when no alarms at all are expected [2]. In their experimental setup Andriessen et al record false alarms that previously have never been seen, neither in the clinical situation nor in our own fetal sheep studies. The reason for this becomes apparent when their way of recording the FECG is scrutinized.

### FECG lead

**Fig 1:**
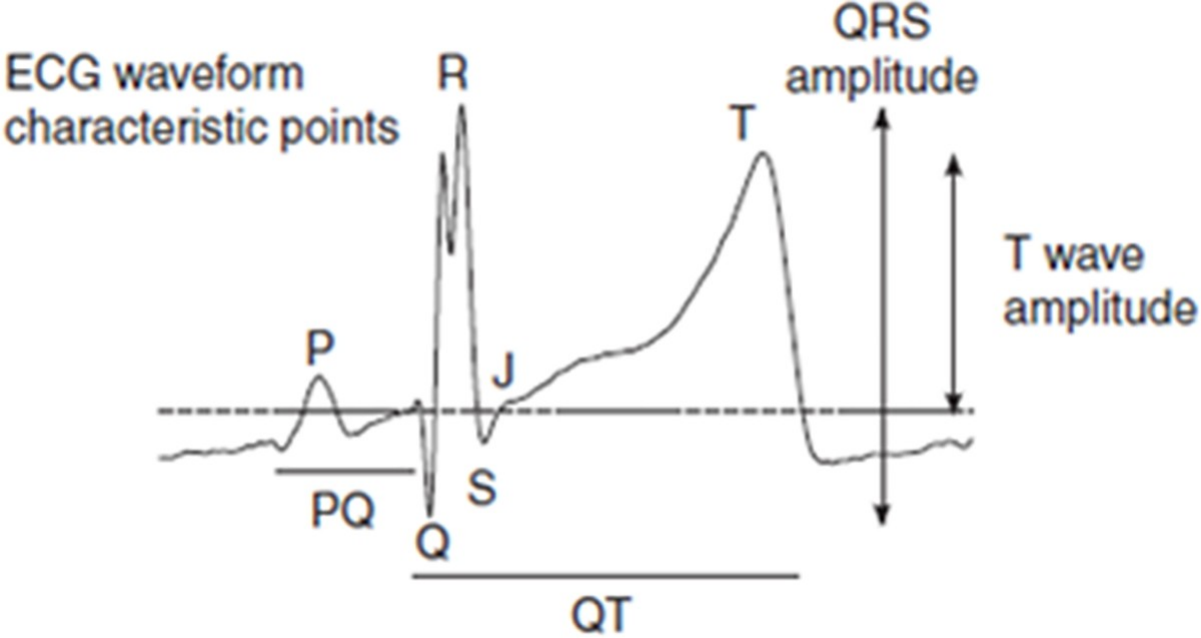
An illustration of the processed negative aVF ECG signal. The ECG sample illustrates QRS-features (Q-wave and split R) that differ from a normal fetal scalp ECG. For further information, see ref. [4].

For clinical use, STAN analysis is based on a unipolar lead [3]. This lead has been used in numerous clinical trials validating STAN, and it has proven to detect the T-vector properly. In their assessment of STAN, Andriessen at al use an assumed negative aVF lead with the assumption that it will reflect the FECG in the same way as the unipolar scalp lead. So in their sheep preparation they record lead I and II of the fetal lamb and use these signals to compute the negative aVF lead. In theory the method may seem reasonable. However, before using the obtained signal for validation of STAN, the signal itself should be validated. No such validation is presented. Neither do they provide any example in the paper of the FECG signal that is the result of their maneuvers, but judging from what is presented in [4] (see Fig 1), an ECG with a split R-peak, their method is flawed. Probably they fail to record the leads I and II properly in utero. Obviously the signal used for disqualification of STAN is itself not qualified to properly represent the fetal scalp lead signal that STAN is designed for.

A major difference as compared to the STAN FECG lead is the split R disabling its use as a trigger point. Instead, the use of the–aVF lead requires Q to be used as a trigger point for FECG averaging and further signal processing [4]. The problem is that STAN algorithms operate using the R or the S waves and not the Q. For the same reason as the authors have found the–aVF- R useless for FECG waveform sampling, STAN will reject the waveform presented and will not calculate ST data.

Another reason may be that the signal amplitude does not comply with the set standards of STAN and the clinically used FECG. The authors have not provided the details of their lead configuration. In fact, the reference paper on the experimental methodology describes the use of chest (precordial) leads with the aim to record the fetal heart rate [5]. The authors of the present paper were using leads I and II constructed from the chest electrodes with undefined position on the fetal chest. Another problem with the present paper is related to the choice of gestational age of the experimental setup and the selection of preparations.

### Gestational age and ST changes

This is a fundamental issue insofar as 105 days old fetuses will not have the same reactivity features as the term fetus. However, the ischemia/asphyxia model will elicit a reaction consisting of an initial increase in T-wave amplitude lasting 12 minutes, the period when fetal blood pressure is increased [2]. A fetus unable to respond to cord occlusion will develop negative T. Midgestation fetal lambs exposed to an inflammatory process will display a negative T-wave component [6].

Andriessen et al, using the STAN monitor present a much different pattern of ST changes consisting of very marked negative T waves—(T/QRS ratio average -0.5) as the initial ST reaction to cord occlusion. But in addition, they present data from one of their eight preparations using their own ST analysis software showing the expected T/QRS rise as the initial response to the occlusion, whereas in other preparations no such rise was detected.

However, a more extensive study using the same design reported negative T waves with a T/QRS ratio of >1 as the predominant fetal response to cord occlusion [4]. These T/QRS data are much different from what we have noted and the question is to what extent the difference in FECG lead configuration between preparations may be the reason?

The authors claim that STAN is unable to detect relevant ST changes and instead produces erroneous alarms. The reason for this is not discussed apart from stating that the STAN algorithms may be unable to detect a T wave decrease and there may be alterations in the fetal heart electrical axis.

The STAN algorithms have been developed over a 15 year period starting with analogue signal processing and finishing with patented digital processing features in 1997 (Rosén KG, Samuelsson A. Device for reducing signal noise in a fetal ECG signal. https://patents.google.com/patent/US6658284B1). The STAN FECG analysis features was a key component in the US Food and Drug Administration (FDA) PMA approval process ruling out the possibility of inadequate ST signal processing by STAN.

**Fig 2:**
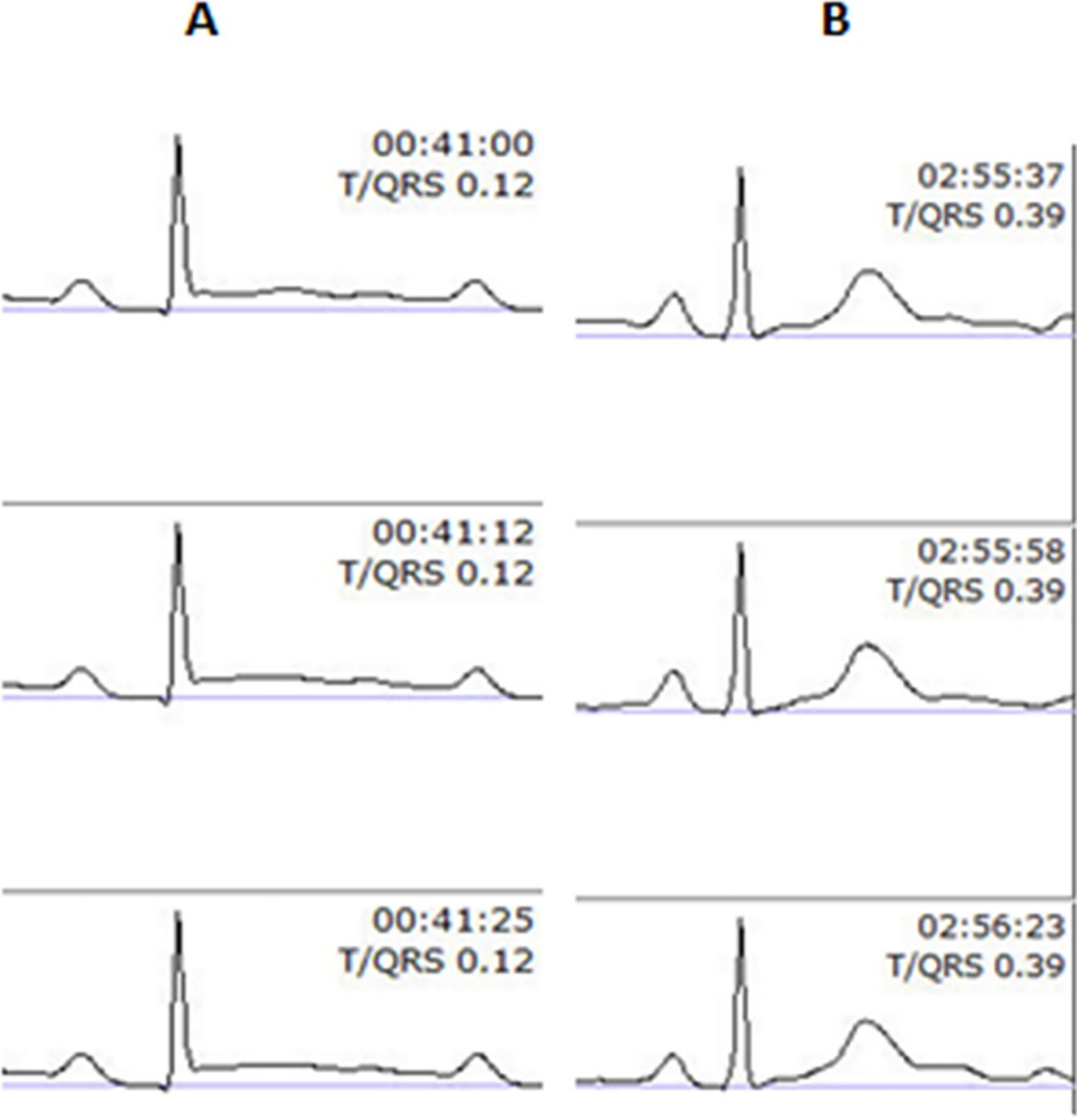
A sequence of FECG recorded by the STAN device. The FECG recorded in connection with the development of metabolic acidosis during labor and illustrates a marked rise in T wave amplitude and T/QRS ratio (from 0.12 to 0.39) during delivery of a term baby. Sequence A provides three averaged FECG samples obtained in first stage of labor and sequence B provides the corresponding data in second stage with active pushing.

What is the possibility of T vector alterations making STAN unable to provide detailed ST data? This is a more complex issue which has been handled using a bipolar FECG lead [7] and the Goldtrace scalp electrode to enable a steady FECG configuration and signal quality throughout delivery. If this bipolar FECG lead would be sensitive to alterations in the electrical axis of the FECG, one would expect concomitant alterations in P and QRS configurations. Fig 2 illustrates the FECG recorded by the STAN device in connection with the development of metabolic acidosis and illustrates a marked rise in T wave amplitude and T/QRS ration (from 0.12 to 0.39) during delivery.

No P or QRS changes can be seen when comparing the **A** sequence obtained during first stage of labor to what was recorded during second stage (**B**).

Furthermore, one of the authors of the Andriessen paper (R Vullings) in his thesis stated that the STAN bipolar lead configuration “was not far from optimal” [8].

## Conclusions

The data presented by Andriessen et al. are of no avail for the evaluation of the ability of the STAN technology to detect fetal hazards. Their use of a calculated negative aVF lead based on signals from an unidentified positioning of fetal chest electrodes, instead of a unipolar scalp lead or precordial leads distorts the configuration of the ECG-complex and creates multiple problems. Most importantly, the split R-wave is rejected by the STAN equipment as a trigger point and instead produces false alarms. The deformation of the R-wave also invalidates calculation of the T/QRS ratio.

To question a proven technology is fully accepted but those attempting would be asked to argue along fully validated data and related analysis including questioning of their own data.
